# Supplementary material for: Effects of Benzodiazepine Exposure on Real-World Clinical Outcomes in Individuals at Clinical High-Risk for Psychosis
Source: medRxiv. 2023 Aug 16:2023.08.15.23294108. Preprint. [Version 1] doi: 10.1101/2023.08.15.23294108 (PMC10462200; doi:10.1101/2023.08.15.23294108)
Supplement: Supplement 1 [file media-1.pdf]

SUPPLEMENTAL DATA

TITLE: Effects of Benzodiazepine Exposure on Clinical Outcomes in Individuals at Clinical High-Risk for Psychosis

AUTHORS: Livingston NR, De Micheli A, McCutcheon R, et al.

Supplementary Table 1. Details of Benzodiazepine Exposures Included in Analysis (n = 105)

Supplementary Table 2. Additional Sensitivity Analyses in the Propensity Score Matched Sample

Supplementary Table 1. Details of Benzodiazepine Exposures Included in Analysis (n = 105)

		Count (%)
**BDZ name**		
	Clonazepam	16 (15.1)
	Lorazepam	12 (11.3)
	Diazepam	16 (15.1)
	Temazepam	1 (0.9)
	Zopiclone/Zolpidem	41 (38.7)
	Alprazolam	1 (0.9)
	Bromazepam	1 (0.9)
	Clonazepam + Lorazepam	1 (0.9)
	Clonazepam + Diazepam	3 (2.8)
	Clonazepam + Zopiclone	7 (6.6)
	Lorazepam + Diazepam	1 (0.9)
	Lorazepam + Zopiclone	1 (0.9)
	Diazepam + Zopiclone	4 (3.8)
	Bromazepam + Zopiclone	1 (0.9)
**Reason for BDZ exposure**		
	Anxiety	17 (16.0)
	Sedation	3 (2.8)
	Agitation	13 (12.3)
	Sleep	59 (55.7)
	Not known	13 (12.3)
		**Mean (± SD)**
**Total number of BDZ exposure (days)**		18.5 (25.6)

BDZ: Benzodiazepine

Supplementary Table2. Additional Sensitivity Analyses in the Propensity Score Matched Sample

		Transition to Psychosis	Psychiatric Hospital Admission	Home Visit	A&E Attendance
**≥ 3 total days of BDZ (n = 89 per group)**					
	HR (95% CI), *P*	1.67 (0.89-3.11), .11	1.67 (0.79-3.53), .18	1.38 (0.86-2.23), .18	1.23 (0.75-2.18), .37
	Number of events; BDZ exposed vs. unexposed	22 vs. 18	16 vs. 12	37 vs. 31	33 vs. 22
**≥ 7 total days of BDZ (n = 66 per group)**					
	HR (95% CI), *P*	1.61 (0.75-3.45), .22	0.82 (0.32-2.12), .69	1.65 (0.94-2.89), .08	1.11 (0.56-2.19), .77
	Number of events; BDZ exposed vs. unexposed	11 vs. 7	7 vs. 4	24 vs. 12	16 vs. 10
**Removing non-benzodiazepine hypnotics (n = 75 per group)**					
	HR (95% CI), *P*	0.91 (0.47-1.77), .79	1.41 (0.58-3.41), .44	1.47 (0.87-2.49), .15	2.08 (1.11-3.93), **.02**
	Number of events; BDZ exposed vs. unexposed	18 vs. 17	13 vs. 8	41 vs. 28	31 vs. 16

A&E: Accident & Emergency; BDZ: Benzodiazepine
